# Financial stress as a mediator of the association between maternal childhood adversity and infant birth weight, gestational age, and NICU admission

**DOI:** 10.1186/s12889-023-15495-0

**Published:** 2023-03-30

**Authors:** David W. Sosnowski, Alejandra Ellison-Barnes, Joan Kaufman, Cathrine Hoyo, Susan K. Murphy, Raquel G. Hernandez, Joddy Marchesoni, Lauren M. Klein, Sara B. Johnson

**Affiliations:** 1 Department of Mental Health, Johns Hopkins Bloomberg School of Public Health, Baltimore, USA; 2 Department of Medicine, Johns Hopkins School of Medicine, Baltimore, USA; 3 Center for Child and Family Traumatic Stress, Kennedy Krieger Institute, Baltimore, USA; 4 Department of Psychiatric and Behavioral Sciences, Johns Hopkins School of Medicine, Baltimore, USA; 5 Department of Biological Sciences and Center for Human Health, North Carolina State University, Raleigh, USA; 6 Department of Obstetrics and Gynecology, Duke University Medical Center, Durham, USA; 7 Johns Hopkins All Children’s Hospital, Baltimore, USA; 8 Department of Pediatrics, Johns Hopkins School of Medicine, Baltimore, USA; 9 Department of Population, Family & Reproductive Health, Johns Hopkins Bloomberg School of Public Health, Baltimore, USA

**Keywords:** Childhood trauma, Poverty, Obstetrics, Infant

## Abstract

**Background:**

To examine whether financial stress during pregnancy mediates the association between maternal exposure to adverse childhood experiences (ACEs) and three birth outcomes (i.e., gestational age, birth weight, and admission to the neonatal intensive care unit [NICU]).

**Methods:**

Data were obtained from a prospective cohort study of pregnant women and their infants in Florida and North Carolina. Mothers (n = 531; *M*_*age at delivery*_ = 29.8 years; 38% Black; 22% Hispanic) self-reported their exposure to childhood adversity and financial stress during pregnancy. Data on infant gestational age at birth, birth weight, and admission to the NICU were obtained from medical records within 7 days of delivery. Mediation analysis was used to test study hypotheses, adjusting for study cohort, maternal race, ethnicity, body mass index, and tobacco use during pregnancy.

**Results:**

There was evidence of an indirect association between maternal exposure to childhood adversity and infant gestational age at birth (*b* = -0.03, 95% CI = -0.06 – -0.01) and infant birth weight (*b* = -8.85, 95% CI = -18.60 – -1.28) such that higher maternal ACE score was associated with earlier gestational age and lower infant birth weight through increases in financial distress during pregnancy. There was no evidence of an indirect association between maternal exposure to childhood adversity and infant NICU admission (*b* = 0.01, 95% CI = -0.02–0.08).

**Conclusions:**

Findings demonstrate one pathway linking maternal childhood adversity to a potentially preterm birth or shorter gestational age, in addition to low birth weight at delivery, and present an opportunity for targeted intervention to support expecting mothers who face financial stress.

## Background

Adverse birth outcomes, including low birth weight and preterm birth, are a serious public health concern given their association with short- and long-term negative health consequences for mothers and children [1]. Short gestation (i.e., < 37 weeks) and low birth weight (i.e., < 2,500 g) accounted for an estimated 17% of all infant deaths in the United States in 2019 [2]. Additionally, the effects of preterm birth can be long-lasting, with one study demonstrating lower educational attainment and IQ, and more neurosensory impairments, among young adults born at very-low birth weight (< 1,500 g) compared to normal weight controls [3]. Researchers have identified numerous prenatal and perinatal factors (e.g., lack of social support, poor maternal physical health) that contribute to adverse birth outcomes [4]. Recent evidence suggests maternal exposure to adverse childhood experiences (ACEs) also contributes to these adverse outcomes, including preterm birth, low birth weight, and neonatal intensive care unit (NICU) admission [5–7]; however, the mechanism(s) linking maternal ACEs to birth outcomes remain unclear.

Researchers have proposed various conceptual models to explain how maternal exposure to childhood adversity may influence later risk for adverse birth outcomes. According to the pathway perspective of life course health proposed by Hertzman [8], early adversities place individuals on life course trajectories that expose them to future adversities such as academic and financial difficulties. Consistent with this perspective, maternal ACEs are associated with increased financial strain during adulthood via decreased education, employment, and income potential [9]. These early experiences, in combination with contemporary circumstances in adulthood, may indirectly confer risk for adverse birth outcomes and negatively affect offspring. From a biological perspective, inflammation has been proposed as an intermediary linking maternal experiences of adversity (before and during pregnancy) to fetal and infant development [10–12]. Indeed, a line of research has demonstrated that psychological stress, for example, negatively impacts immune function (e.g., increased inflammation), which is associated with preterm birth [11]; maternal exposure to ACEs, specifically, has been linked to increases in inflammatory markers (e.g., CRP, IL-6), which pose risk for adverse birth outcomes and infant psychiatric risk [10]. Despite the evidence around these and other potential mechanisms (e.g., genetic, epigenetic), most studies of ACEs and adverse birth outcomes have focused on direct associations [5–7] and few have tested mechanistic pathways [13, 14]. One pathway that may explain the association between maternal exposure to ACEs and adverse birth outcomes is financial stress during pregnancy.

Financial stressors (e.g., difficulty paying bills, fear of losing home or job) during pregnancy are common in the United States. From 2013 to 2018, 60% of peripartum mothers reported healthcare unaffordability and 54% reported general financial stress [15]. These rates are alarming given evidence linking financial stress during pregnancy to adverse birth outcomes, in part through increased pregnancy stress and depressive symptoms [16]. It is possible that maternal exposure to ACEs is indirectly associated with adverse birth outcomes through financial stress during pregnancy since this may limit mothers’ access to care and/or engagement with prenatal care providers to ensure a safe and healthy pregnancy. Alternatively, mothers may engage in unhealthy behaviors (e.g., smoking) or lack necessary social support to cope with financial stressors during pregnancy, further increasing risk for adverse birth outcomes.

The goal of the present study was to test whether maternal exposure to ACEs is indirectly associated with three birth outcomes (infant birth weight, gestational age, and NICU admission) through financial stress during pregnancy. We hypothesized that a higher maternal ACE score would be associated with greater financial stress during pregnancy, which would be associated with a greater likelihood of NICU admission, earlier gestational age at birth, and lower infant birth weight.

## Methods

### Study sample

Pregnant women (n = 577) were enrolled between April 2018-March 2020 in two cohorts—the Stress and Health in Pregnancy (SHIP) cohort in North Carolina and the Prospective Research on Early Determinants of Illness and Children’s Health Trajectories (PREDICT) cohort in Florida. Both cohorts enrolled from university-affiliated obstetric clinics. Women were eligible if the mother was > 18 years old, spoke and read English (PREDICT), or English or Spanish (SHIP), and planned to deliver at the study-affiliated hospital. Women were ineligible if the fetus had a known congenital anomaly or chromosomal abnormality, or if the mother had HIV, Hepatitis C, or Hepatitis B. Average gestational age at enrollment was 20.8 weeks (SD = 6.9) for SHIP and 24.6 weeks (SD = 6.3) for PREDICT. After enrollment, women completed questionnaires about demographics and health behaviors and provided obstetric, medical, and social histories. Maternal obstetric and infant delivery records were abstracted following delivery. The SHIP study was approved by the Institutional Review Board (IRB) at North Carolina State University, and the PREDICT study was approved by the IRB at Johns Hopkins All Children’s Hospital. Mothers provided written informed consent for themselves and their children. In the present study, 12 mothers who gave birth to multiple children (e.g., twins; n = 24) were removed from analyses, in addition to eight cases due to intrauterine fetal demise and 14 mothers who reported a race other than Black or white. Thus, the analytic sample for this study included 531 mother-infant dyads.

### Measures

#### Adverse childhood experiences

Mothers reported on their ACEs during the first study visit (i.e., 2nd trimester) using the 10-item measure developed by Felitti et al. [17] that assesses childhood physical, emotional, and sexual abuse and emotional and physical neglect. Scores were summed to create a total ACE score (range: 0–10).

#### Financial stress

Financial stress was also measured during the first study visit using the 6-item Financial Stress Index [18], [38], which assesses the frequency of financial stressors in the last three months (e.g., difficulty paying bills, fears of losing home/job). Items are Likert scaled from 0 (never) to 4 (always) and summed (range: 0 to 24) with higher scores indicating more financial stress.

#### Birthweight, gestational age, and NICU stay

Infant birthweight, gestational age at birth, and NICU admission during the initial delivery hospitalization stay were preferentially extracted from hospital delivery records (admission to the NICU following delivery discharge was not included). In the few cases where these data were missing (n = 9), maternal reports of gestational age were obtained at six weeks postpartum.

#### Covariates

Covariates included study cohort, maternal pre-pregnancy body mass index (BMI; from obstetric records), self-reported tobacco use during the pregnancy (yes/no), race (Black/African American [including those who described themselves as Black/African American and one or more additional racial group], or white), and ethnicity (Hispanic/Latina or not Hispanic/Latina).

### Analytic methods

All analyses were conducted in R version 4.1.3 [19]. Prior to analyses, data were inspected for normality and outliers. Data were approximately normally distributed, and possible outliers were detected due to very low birth [20] (i.e., birth weight < 1500 g; n = 3) and macrosomia [21] (i.e., birth weight > 4500 g; n = 6). Analyses were run without these participants to determine how robust findings were to removal of outliers. Next, we inspected missing data. There were minimal missing data across variables included in the analytic models: maternal ACEs (6.2% of participants), financial stress (9%), BMI (2.8%), and ethnicity (0.4%); infant’s birth weight (7.9%), gestational age (7.9%), and NICU status (13.6%). Results from Little’s MCAR test were significant (*p* < 0.001), suggesting that data were not missing completely at random; however, visual inspection of the patterns of data did not reveal substantial differences between those with and without data on these variables. Given this, and the minimal amount of missing data, we assumed data to be missing at random and imputed missing data using pairwise deletion for our binary outcome (NICU admission) and full information maximum likelihood estimation for our continuous outcomes (gestational age at birth and birth weight).

Mediation was tested using the product method [22], which requires significant relationships between the independent variable and mediator, and mediator and outcome variables, in order for a variable to be defined as a mediator. In addition, all confounders of the exposure-outcome and mediator-outcome relationships must be controlled for to have an accurate causal interpretation [23]. All mediation models were run using the R package *lavaan* [24]. Diagonally weighted least squares estimation was used for our binary outcome, with a probit link function, and maximum likelihood estimation was used for the continuous outcomes. Bootstrapped standard errors were obtained in all models using 5,000 draws, and statistical significance was set at a threshold of p < 0.05.

## Results

Demographic and study characteristics are shown in Table 1. Mothers were approximately 30 years of age at the time of delivery (*M* = 29.80, *SD* = 5.75); approximately half reported having public insurance at study enrollment; the majority (62%) of mothers reported their race as white; and 22% reported their ethnicity as Hispanic. Notably, most mothers were overweight, with an average BMI of 29.73, and although 31 mothers in the SHIP cohort self-reported tobacco use during pregnancy, all mothers in the PREDICT cohort denied tobacco use during pregnancy. Number of ACEs reported ranged from 0 to 8 (Interquartile range [IQR] = 0–2), with similar exposure to childhood adversity across cohorts. On average, mothers in both cohorts reported low amounts of financial stress during pregnancy (*M* = 5.75, *SD* = 5.41); average infant gestational age at birth was 38.63 weeks, which is considered early term, and 12% of babies were admitted to the NICU.

**Table 1:** Demographic and Study Characteristics

Demographics	Full Sample (n = 531)	SHIP (n = 317)	PREDICT (n = 214)
Maternal Age, M (SD)	29.80 (5.75)	29.13 (6.19)	30.87 (4.79)
Race, N (% Black)	203 (38)	172 (54)	31 (14)
Hispanic/Latina, N (%)	119 (22)	104 (33)	15 (7)
Tobacco Use, N (%)	31 (6)	31 (10)	0 (0)
Maternal BMI, M (SD)	29.73 (8.89)	31.41 (9.40)	27.27 (7.44)
Insurance, N (% Public)	260 (52)	206 (72)	54 (26)
Study Variables			
ACEs, Median (IQR)	0 (0–2)	0 (0–2)	0 (0–2)
Financial Stress, M (SD)	5.75 (5.41)	7.19 (5.75)	3.91 (4.30)
Birth Weight, M (SD)	3264.8 (583.47)	3183.29 (613.39)	3378.68 (519.28)
Gestational Age, M (SD)	38.63 (2.26)	38.25 (2.66)	39.16 (1.39)
NICU Admission, N (%)	57 (12)	47 (17)	10 (6)

*Note*. Mother age reflects age at delivery. Race was coded as Black or white. Birth weight is measured in grams and gestational age is measured in weeks. BMI = body mass index; NICU = neonatal intensive care unit; ACEs = adverse childhood experiences; M = mean; SD = standard deviation; IQR = interquartile range.

### Mediation analyses

We conducted a series of mediation analyses to test the direct and indirect association between maternal exposure to ACEs and several birth outcomes through financial stress during pregnancy. Table 2 contains a summary of findings for the direct, indirect, and total effects from each model, including after the removal of outliers based on birth weight (n = 9).

**Table 2:** Results from Mediation Models

	NICU	NICU (No outliers)	Gestational Age	Gestational Age (No outliers)	Birth Weight	Birth Weight (No outliers)
	*b* [95% CI]	*b* [95% CI]	*b* [95% CI]	*b* [95% CI]	*b* [95% CI]	*b* [95% CI]
Direct Effect	− 0.03 [-0.16, 0.05]	− 0.17 [-0.25, 0.12]	0.03 [-0.04, 0.11]	0.04 [-0.03, 0.12]	14.48 [-17.91, 47.00]	13.22 [-14.98, 40.06]
Indirect Effect	0.01 [-0.02, 0.08]	0.14 [-0.15, 0.19]	**− 0.03 [-0.06, − 0.01]**	**− 0.03 [-0.06, − 0.01]**	**-8.85 [-18.60, -1,28]**	**-7.96 [-17.45, -0.81]**
Total Effect	− 0.02 [-0.14, 0.06]	− 0.32 [-0.16, 0.05]	0.003 [-0.07, 0.08]	0.01 [-0.06, 0.09]	5.63 [-27.26, 38.19]	5.26 [-24.67, 32.88]

*Note*. Bolded values reflect significant results. NICU = neonatal intensive care unit. CI = confidence interval. Nine outliers removed due to low birth weight (n = 3) and macrosomia (n = 6).

#### Neonatal intensive care unit (NICU) admission

First, we tested whether maternal exposure to ACEs was associated with NICU admission directly, as well as indirectly through financial stress during pregnancy. Higher maternal ACE score was associated with greater financial stress during pregnancy (*b* = 0.63, 95% CI = 0.39–0.92), though there was no evidence of an association between maternal ACE score and NICU admission (*b* = -0.03, 95% CI = -0.16–0.05) or financial stress and NICU admission (*z* = 0.01, 95% CI = -0.03–0.19). There also was no evidence of an indirect association between maternal ACE score and NICU admission through financial stress (*b* = 0.01, 95% CI = -0.02–0.08). These results were robust to removal of outliers.

#### Gestational age

Next, we tested whether maternal exposure to ACEs was directly associated with gestational age at birth, and indirectly through financial stress during pregnancy. Higher maternal ACE score was associated with more financial stress (*b* = 0.64, 95% CI = 0.38–0.90), though there was no evidence of an association between maternal ACE score and gestational age (*b* = 0.03, 95% CI = -0.40–0.11). Mothers’ financial stress during pregnancy was inversely associated with gestational age at birth (*b* = -0.05, 95% CI = -0.08 – -0.02), and there was an indirect association between maternal ACE score and gestational age through financial stress (*b* = -0.03, 95% CI = -0.06 – -0.01) such that a higher ACE score was associated with earlier gestational age through increased financial stress during pregnancy. These results were robust to removal of outliers.

#### Birth weight

Lastly, we tested whether maternal exposure to ACEs was directly associated with birthweight, and indirectly associated through financial stress during pregnancy. Higher maternal ACE score was associated with greater financial stress during pregnancy (*b* = 0.64, 95% CI = 0.37–0.92), though there was no association between maternal ACEs and birth weight (*b* = 14.48, 95% CI = -17.91–47.00). Mother’s financial stress during pregnancy was inversely associated with infant birth weight (*b* = -13.86, 95% CI = -25.05 – -2.39), and there was evidence of an indirect association between maternal ACEs and birth weight through financial stress (*b* = -8.85, 95% CI = -18.60 – -1.28). These results were robust to removal of outliers.

## Discussion

We undertook an analysis to test the hypothesis that maternal ACEs scores increased the risk of financial stress during pregnancy, which in turn increased the likelihood of NICU admission, earlier gestational age at birth, and lower infant birth weight. This study demonstrated that maternal exposure to ACEs is indirectly associated with earlier infant gestational age at birth and lower infant birth weight through increased financial stress during pregnancy. There was no evidence to suggest that maternal exposure to ACEs is directly or indirectly associated with infant probability of a stay in the NICU. These findings elucidate one pathway through which intergenerational stress affects children at the earliest stages of development and present an opportunity for researchers and policy makers to improve prenatal care in ways that are tenable and affordable.

Contrary to our findings, extant evidence supports a direct association between maternal exposure to ACEs and numerous birth outcomes, including low birth weight and early gestational age [14, 25–27]. However, these published findings may not be comparable to our data as the median number of ACEs reported by mothers in the present study was low (0; IQR = 0–2), and prior studies note associations with adverse birth outcomes at high (e.g., 3+, 6+) levels of adversity [6, 28]. It is possible that this direct association is observed only at high levels of adversity that affect maternal functioning longer throughout the lifecourse. Regarding financial stress, prior research demonstrates a link between financial strain during pregnancy and low infant birth weight [16]. In our study, we found direct associations between financial stress and both infant gestational age and weight at birth, but not NICU admission. This may be due, in part, to how we operationalized financial stress. Most studies testing the association between financial stress and birth outcomes use indicators of socioeconomic resources such as employment status, annual income, and education [25, 26, 29]. Here, we operationalized financial stress based on its perceived impact using five indicators reflecting the severity of specific material hardships (e.g., difficulty paying bills), which may provide more sensitivity to detect mothers experiencing financial strain.

Our finding that financial stress during pregnancy mediated the association between maternal exposure to ACEs and earlier infant gestational age and weight at birth underscores the intergenerational impact of ACEs and immediate impact of financial strain on children. Moreover, this presents an opportunity to intervene during a critical period of prenatal development. Evidence suggests that financial stress during pregnancy negatively affects birth outcomes through lack of affordability and subsequent engagement in prenatal care [15] and/or through increased psychosocial stressors, including anxiety and depression [16]. All mothers in the current study had access to, and were receiving, prenatal care, so it is likely that ACEs indirectly contributed to earlier gestational age through the various stressors that come with living under financial stress. Although socioeconomic factors (e.g., employment, annual income) may be difficult to adjust in the short term to relieve financial stress, options exist to improve mothers’ psychosocial wellbeing and prevent adverse birth outcomes.

There are several empirically supported means through which researchers and policy makers can support mothers experiencing financial stress and associated stressors during pregnancy. The Family Foundations [30] intervention is one way in which families experiencing financial stress can be supported, particularly given that short-term changes in socioeconomic status may be infeasible. Family Foundations is a universal, evidence-based intervention, delivered to first-time parents during and after pregnancy to increase cooperative co-parenting, that has been associated with better parent mental health (including perceived financial stress), birth outcomes (e.g., birth weight) [31], and parent-child relations [30]. By enhancing parents’ communication and support, mothers may experience less psychosocial stress, including stress related to finances, thus reducing the risk of adverse birth outcomes.

Alternative measures for supporting both first-time and existing parents include (but are not limited to) child tax credits, extension of Medicaid coverage from 60 days to a full year post-partum [32], paid parental leave, and guaranteed income programs. Evidence suggests that child tax credits, for example, are an effective anti-poverty strategy, reducing food insecurity in the short-term [33] and improving maternal mental health, specifically among single mothers [34]. Although empirical evidence is limited in the US, guaranteed income programs also represent an alternative approach to assisting expecting mothers. For example, the Abundant Birth Project [35] in San Francisco seeks to provide an income supplement to Black and Pacific Islander pregnant women in an effort to decrease stress and improve infant and mother outcomes. The evaluation study of this project is currently underway. Combined with evidence-based interventions, these policies provide multiple angles by which to reduce the financial burden and associated stressors for expecting mothers, which in turn has the potential to improve birth outcomes and infant health.

The present study had several strengths, including a racially and ethnically diverse sample of expecting mothers and temporal ordering of events that aids in establishing causation. There are, however, several limitations to consider when interpreting these results. First, we were not able to adjust for all possible confounders, namely mother’s childhood socioeconomic status, which presents a major potential confounder of the exposure-mediator and exposure-outcome relationships [36]. Second, overall levels of ACEs (and financial stress) were low-to-moderate in this sample; the ACE measure included in this study also did not include racialized experiences and other relevant adversities. Recent evidence shows that, among Black mothers, additional ACEs (e.g., perceived racism, neighborhood safety) contribute to poor health outcomes (e.g., depression) [37]. It is possible that the present associations are underestimated due to exclusion of these experiences for mothers from minoritized racial and ethnic groups. This study also was limited in that mothers self-reported on tobacco use throughout pregnancy and social desirability bias could have affected our findings. Similarly, our results are subject to shared-method bias with mothers reporting on both their ACEs and financial stress during pregnancy.

## Conclusions

Our study findings suggest that maternal exposure to ACEs increases risk for adverse birth outcomes (i.e., earlier gestational age, low birth weight) through increases in financial stress during pregnancy. Existing interventions and policies can be implemented to better serve mothers exposed to childhood adversity who may be at risk for financial strain during pregnancy. Future studies that focus on specific aspects of financial strain (e.g., housing insecurity, food insecurity) and mechanisms (e.g., epigenetic changes) linking financial strain to these adverse outcomes will paint a clearer picture of the intergenerational transmission of stress and ways to improve the health of children at the earliest stages of development.

## Data Availability

The datasets generated and/or analyzed during the current study are not publicly available due to reasons of sensitivity but are available from the corresponding author on reasonable request.

## List of abbreviations

ACEs: Adverse Childhood Experiences
BMI: Body Mass Index
CI: Confidence Interval
IQR: Interquartile Range
NICU: Neonatal Intensive Care Unit
