# Correction: Financial stress as a mediator of the association between maternal childhood adversity and infant birth weight, gestational age, and NICU admission

**DOI:** 10.1186/s12889-023-15783-9

**Published:** 2023-05-05

**Authors:** David W. Sosnowski, Alejandra Ellison-Barnes, Joan Kaufman, Cathrine Hoyo, Susan K. Murphy, Raquel G. Hernandez, Joddy Marchesoni, Lauren M. Klein, Sara B. Johnson

**Affiliations:** 1 Department of Mental Health, Johns Hopkins Bloomberg School of Public Health, Baltimore, USA; 2 Department of Medicine, Johns Hopkins School of Medicine, Baltimore, USA; 3 Center for Child and Family Traumatic Stress, Kennedy Krieger Institute, Baltimore, USA; 4 Department of Psychiatric and Behavioral Sciences, Johns Hopkins School of Medicine, Baltimore, USA; 5 Department of Biological Sciences and Center for Human Health, North Carolina State University, Raleigh, USA; 6 Department of Obstetrics and Gynecology, Duke University Medical Center, Durham, USA; 7 Johns Hopkins All Children’s Hospital, Baltimore, USA; 8 Department of Pediatrics, Johns Hopkins School of Medicine, Baltimore, USA; 9 Department of Population, Family & Reproductive Health, Johns Hopkins Bloomberg School of Public Health, Baltimore, USA

**Correction: BMC Public Health 23, 606 (2023)**

**https://doi.org/10.1186/s12889-023-15495-0**

The original publication of this article [1] contained an in-text error in how the authors described the measure of financial stress. The incorrect and correct information is listed below. The citation for Essex et. Al [38] has also been added.

**Incorrect**

Financial stress was also measured during the first study visit using the 5-item Financial Stress Index [18], which assesses the frequency of financial stressors in the last three months (e.g., difficulty paying bills, fears of losing home/job). Items are Likert scaled from 0 (never) to 5 (always) and summed (range: 0 to 25) with higher scores indicating more financial stress.

**Correct**

Financial stress was also measured during the first study visit using the **6-item** Financial Stress Index [18], [38], which assesses the frequency of financial stressors in the last three months (e.g., difficulty paying bills, fears of losing home/job). Items are Likert scaled from **0 (never) to 4 (always)** and summed **(range: 0 to 24)** with higher scores indicating more financial stress.
