# Correction to: Prevalence and associated factors of smoking among chinese adolescents: a school-based cross-sectional study

**DOI:** 10.1186/s12889-023-15921-3

**Published:** 2023-06-28

**Authors:** Bingliang Lin, Xin Liu, Wenlong Lu, Xiaobing Wu, Yanyan Li, Ziyang Zhang, Rongyin Fu, Luge Zhang, Jingfan Xiong

**Affiliations:** 1 Shenzhen Center for Chronic Disease Control, No.2021 Buxin Road, Luohu District, Shenzhen, 518001 China; 2 Chinese Center for Disease Control and Prevention, No.27 Nanwei Road, Xicheng District, Beijing, 100032 China

**Correction to**: ***BMC Public Health*23, 669 (2023)**

10.1186/s12889-023-15565-3

The original publication of this article contained 2 errors:

1. The affiliation “Shenzhen Center for Chronic Disease Control” was not shown for Bingliang Lin.
2. The acknowledgement section did not corresponding with the funding section.

The original article has been updated.

